# Mechanical ventilation in patients in the intensive care unit of a general university hospital in southern Brazil: an epidemiological study

**DOI:** 10.6061/clinics/2016(03)05

**Published:** 2016-03

**Authors:** Léa Fialkow, Maurício Farenzena, Iuri Christmann Wawrzeniak, Janete Salles Brauner, Sílvia Regina Rios Vieira, Alvaro Vigo, Mary Clarisse Bozzetti

**Affiliations:** I Universidade Federal do Rio Grande do Sul, Departamento de Medicina Interna; II Departamento de Medicina Social, Porto Alegre/, RS, Brazil; III Hospital de Clínicas de Porto Alegre, Divisão de Cuidados Intensivos, Porto Alegre/, RS, Brazil; IV Universidade Federal do Rio Grande do Sul, Instituto de Matemática, Departamento de Estatística, Porto Alegre/, RS, Brazil

**Keywords:** Acute Respiratory Failure, Mechanical Ventilation, Mortality, Risk Factors, Epidemiology

## Abstract

**OBJECTIVES:**

To determine the characteristics, the frequency and the mortality rates of patients needing mechanical ventilation and to identify the risk factors associated with mortality in the intensive care unit (ICU) of a general university hospital in southern Brazil.

**METHOD:**

Prospective cohort study in patients admitted to the ICU who needed mechanical ventilation for at least 24 hours between March 2004 and April 2007.

**RESULTS:**

A total of 1,115 patients admitted to the ICU needed mechanical ventilation. The mortality rate was 51%. The mean age (± standard deviation) was 57±18 years, and the mean Acute Physiology and Chronic Health Evaluation II (APACHE II) score was 22.6±8.3. The variables independently associated with mortality were (i) conditions present at the beginning of mechanical ventilation, age (hazard ratio: 1.01; *p*<0.001); the APACHE II score (hazard ratio: 1.01; *p*<0.005); acute lung injury/acute respiratory distress syndrome (hazard ratio: 1.38; *p*=0.009), sepsis (hazard ratio: 1.33; *p*=0.003), chronic obstructive pulmonary disease (hazard ratio: 0.58; *p*=0.042), and pneumonia (hazard ratio: 0.78; *p*=0.013) as causes of mechanical ventilation; and renal (hazard ratio: 1.29; *p*=0.011) and neurological (hazard ratio: 1.25; p=0.024) failure, and (ii) conditions occurring during the course of mechanical ventilation, acute lung injuri/acute respiratory distress syndrome (hazard ratio: 1.31; *p*<0.010); sepsis (hazard ratio: 1.53; *p*<0.001); and renal (hazard ratio: 1.75; *p*<0.001), cardiovascular (hazard ratio: 1.32; *p*≤0.009), and hepatic (hazard ratio: 1.67; *p*≤0.001) failure.

**CONCLUSIONS:**

This large cohort study provides a comprehensive profile of mechanical ventilation patients in South America. The mortality rate of patients who required mechanical ventilation was higher, which may have been related to the severity of illness of the patients admitted to our ICU. Risk factors for hospital mortality included conditions present at the start of mechanical ventilation conditions that occurred during mechanical support.

## INTRODUCTION

Acute respiratory failure (ARF) is a common cause of admission to the intensive care unit (ICU) that occurs for several reasons, including pulmonary disease, neuromuscular disease, shock and the need for airway protection or temporary respiratory support after major surgery. For patients with ARF, mechanical ventilation (MV) is the cornerstone of management 1,2. Patients admitted to ICUs who need MV are expected to have higher mortality rates compared with those who do not require respiratory support 3.

Despite advances in the management of ARF with MV, mortality has not decreased significantly and costs remain high 4,5,6,7,8,9. Knowledge of the epidemiology of patients requiring MV, including mortality rates and mortality risk factors, may help to improve therapeutic strategies, as might counseling of patients or their relatives 10. Esteban et al. found that mortality depends on (i) factors present at the start of MV, (ii) factors developed during the course of MV, and (iii) factors related to patients' management 10. Other studies have addressed the specific risk factors in such patients, including multiple organ dysfunction, age, the Simplified Acute Physiology Score II (SAPS II), nonpulmonary sources of respiratory failure, and immunosuppression 3, 5,.

Knowledge of the epidemiological profile of patients with ARF requiring MV in Latin America is scarce, particularly in Brazil. In particular, Tomicic et al. 14 explored the characteristics and mortality risk factors of 156 patients who required MV in 19 ICUs in Chile, and Azevedo et al. 15 evaluated 773 adult patients admitted to 45 ICUs in Brazil over a two-month period who required MV for more than 24 hours. Further studies are needed to improve the understanding of patients who require MV in developing countries in which there is limited resource allocation. Therefore, our study aimed to determine the characteristics, the frequency and the mortality rates of patients who needed MV in southern Brazil.

## MATERIALS AND METHODS

### Study design

This prospective cohort study was conducted between March 1^st^, 2004 and April 30^th^, 2007, in the ICU of the Hospital de Clínicas de Porto Alegre, a teaching hospital in the city of Porto Alegre, in southern Brazil. In Porto Alegre and its metropolitan area, the estimated population size is 1,436,123 and 4,063,886 inhabitants, respectively.

The ICU has 24 beds and is assisted by one doctor and nurse assistant for every five patients and by one nurse technician for every two patients during the day. In addition, one physiotherapist and one medical resident aid every ten and two patients, respectively. During the night and on weekends, one doctor and nurse assistant help every ten patients, one nurse technician helps every two patients, and one medical resident helps every ten patients. At the time of the study, the ICU occupancy rate was 90%.

### Data collection

Patients admitted to the ICU who were ≥18 years old were included in the study after completing 24 consecutive hours on either non-invasive MV (NIMV) or invasive MV (IMV). Patients were excluded if they did not complete 24 hours on MV or did not require it. Each patient was followed during the course of MV for a period of 28 days or until death. All information was collected by trained personnel through a standardized questionnaire.

### Study variables

The following information was obtained:

#### Outcome variables

(i) the overall mortality rate, defined as the mortality rate of all patients admitted to the ICU during the period of the study; (ii) the specific mortality rate, defined as the total number of deaths among MV patients during the study period; and (iii) the length of stay (LOS) in the ICU and hospital.

#### Independent variables

Age; gender; the Acute Physiology and Chronic Health Evaluation II (APACHE II) score obtained within the first 24 hours of admission; patient status (clinical or surgical); the modality of respiratory support (NIMV or IMV); the main reason(s) for MV; tracheostomy; and previous health status, including hepatic, renal, cardiovascular, neurological, gastrointestinal and hematologic failure 16. Data were collected regarding events that developed during the MV period, including acute lung injury (ALI)/acute respiratory distress syndrome (ARDS) 17; barotrauma; sepsis; ventilator-acquired pneumonia (VAP) 18; organ failure 16; weaning failure; and use of drugs such as sedatives, analgesics, and vasoactive or neuromuscular blockers. Weaning failure was defined as a need for reintubation and IMV within the first 48 hours after extubation. Data on arterial blood gases, chest X-rays, ventilatory parameters and modes of MV were also recorded.

### Statistical analysis

Data analysis was performed considering factors present at the start and/or occurring over the course of MV. Categorical variables were coded as dummy variables to compare all categories with the one having the lower mortality. A univariate analysis was performed to identify the variables associated with mortality using either the chi-squared (χ^2^) test (categorical variables) or Student's *t* test (continuous variables). Continuous variables were also compared using ANOVA and the *post hoc* Tukey test. A χ^2^ test for linear trends was used to relate mortality rates to the number of organ failures. A Kaplan-Meier analysis was used to determine the probability of survival during MV. A Cox proportional hazards model was conducted using the PHREG procedure from SAS to identify factors independently associated with mortality. In this analysis, the outcome was defined as the time, in days, between the beginning of MV and death. The criteria for variables entering the multivariable model was *p*<0.25 in the univariable models and/or clinical relevance. Variables were excluded from the model one at a time, keeping those significant at 5% in the final model. The statistical analysis was performed using SPSS® version 18.0 and SAS® version 9.2. The analysis was reviewed by a consultant with formal statistical training and experience. His analysis was performed after all data collection and after the analysis had been independently carried out by another investigator.

### Ethics

The study was approved by the hospital's research ethics committee (number: 03-502) with an informed consent waiver. The research team also signed an institutional document ensuring full confidentiality of the patients' records.

## RESULTS

A total of 2,430 patients were admitted to the ICU during the study period, and 1,115 (46%) were included in the study.

Table 1 describes the characteristics of the studied population. The most common reasons for initiation of MV were sepsis (41.8%), shock (37.8%), pneumonia (37%) and ALI/ARDS (15%). The frequency of IMV was 97.7%, which included patients who started on this mode of ventilation at the beginning of the study (92.6%) and those who failed NIMV (5.1%). The rate of weaning failure for IMV was 29.4%.

Regarding NIMV, 7.4% (n=83) patients initiated this mode of ventilatory support, but only 2.3% (n=26) remained on NIMV during the entire course of MV. Therefore, the failure rate of NIMV was 68.7%. The major reasons for the use of NIMV were pneumonia (49%; n=41), sepsis (31%; n=26), ALI/ARDS (17%; n=14), and chronic obstructive pulmonary disease (COPD) (15%; n=13). The rate of NIMV failure was 79% in ALI/ARDS, 61.5% in pneumonia, 73% in sepsis, and 38.5% in COPD. Additionally, 10.4% of the patients needed tracheostomy and 70.1% (782/1115) needed vasopressors.

The ventilator modes and the monitored respiratory variables on days 1, 2 and 3 (mean values) of IMV in all patients as well as in the COPD and ARDS groups in particular are presented in Table 2. Assisted/controlled pressure ventilation was the most commonly used mode of IMV in all groups. Table 3 describes the outcomes among the COPD, ARDS and other patient (non-COPD and non-ARDS) groups. Patients with ARDS presented a longer duration of IMV compared with those with COPD and other patients (12.1±8.7 *vs*. 8.9±5.5 and 9.3±7.3, *p*=0.01). The LOS values in the ICU and hospital were similar among groups.

**Figure 1-:**
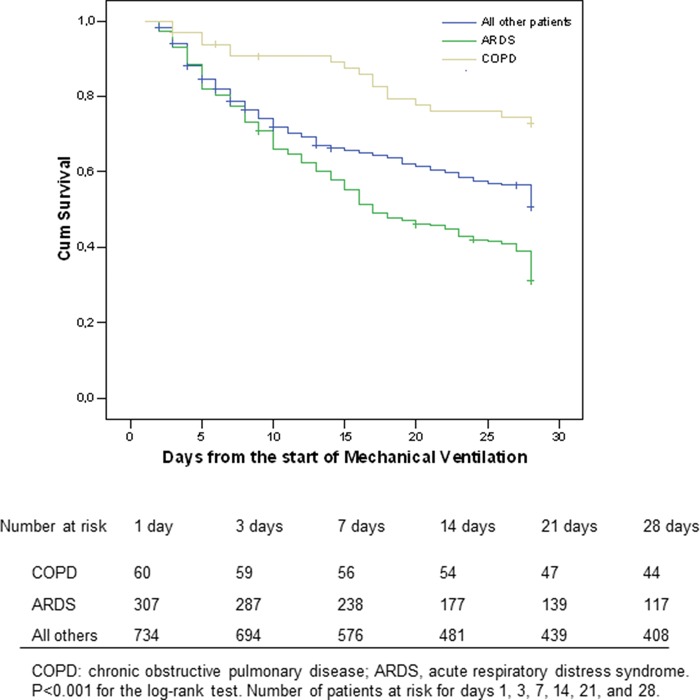
**Figure 1.** Kaplan-Meier curves of the probability of survival over time for mechanical ventilation.

The overall and the specific mortality rates of MV patients were 23% (564/2430) and 51% (564/1115), respectively. Regarding COPD patients in particular, the mortality rate was 26.7% (16/60). In ARDS patients, the mortality rate was 66% (204/307). The mortality rate in patients who developed ARDS during MV (71%; 126/180) was higher, but not significantly different (*p*=0.15), compared with the rate in those with ARDS as the cause of MV (63%; 78/127). Figure 1 presents the survival curves for the ARDS and COPD groups and also for the other studied patients (*p*<0.001).

The main events that occurred during MV were sepsis (n=210; 18.8%), VAP (n=181; 16%), ARDS (n=180; 16%), and barotrauma (n=32; 2.8%). The mortality rates are shown in Table 4; these rates increased as the number of failing organs increased. There was a high percentage of patients with failure of three or more organs in the cohort (45%), especially among patients with ARDS (52%) and in COPD patients (15%). In addition, patients developed the following types of organ failure during the course of MV: renal (n=290; 26%), cardiovascular (n=227; 20.4%), coagulopathic (n=201; 18%), neurological (n=115; 10%) and hepatic (n=86; 7.7%).

The factors independently associated with hospital mortality are illustrated in Table 5. The conditions present at the beginning of MV were age; the APACHE II score; renal and neurological failure; and, as causes of MV, ALI/ARDS and sepsis. The conditions occurring over the course of MV were ALI/ARDS; sepsis; and renal, cardiovascular, and hepatic failure. The following variables were negatively associated with mortality: COPD exacerbation and pneumonia as causes of MV, in addition to VAP.

## DISCUSSION

The main findings of our study were as follows: (i) the hospital mortality rate of patients who required MV was 51% and (ii) the factors associated with increased mortality included conditions present at the start of MV as well as conditions that occurred during mechanical support.

The frequency of MV in our study was in agreement with frequencies described in other publications 3,11. Carson et al. showed that the incidence of MV increased by 11% over 7 years, with a higher burden of comorbidities and fewer discharges to home 8. Additionally, assisted/controlled pressure ventilation was the most commonly used mode of IMV in all groups in the current study and this observation was similar to the results of another study 10.

The rate of mortality in the present study was higher than rates in several multicenter prospective cohort studies, which ranged from 30.7-42% 5,8,10,11,14,15,19. In one Brazilian multicenter study by Azevedo et al., the hospital mortality rate was 42% 15. When comparing the mortality rates in our study with those in the literature, several aspects must be taken into account. These aspects include different methodologies (e.g., inclusion criteria, characteristics of the population) and different severities of illness. Our study specifically showed that APACHE II score, organ failures, ALI/ARDS, sepsis and age were independently associated with hospital mortality. Mortality risk factors have also been the focus of several investigations 3,5,8,10,11,. The high severity of illness in our cohort may have contributed to the elevated mortality rates. In this study, the mean APACHE II score was higher when compared with scores in other studies 5,14. Studies have also evaluated the severity of illness using the SAPS II score, which is difficult to compare across studies 10,11,15. Further evidence that our population had more severe illness was the higher percentage of patients with failure of three or more organs (45%) compared with the percentage in another study, in which 13% of the patients had this condition 13. Other reasons for higher mortality in our study were the reasons for initiation of MV, which included sepsis (41.8%), shock (37.8%), pneumonia (37%) and ALI/ARDS (15%). Azevedo et al. showed that the main causes included pneumonia (27%), neurological failure (19%) and non-pulmonary sepsis (12%) 15. In addition, Esteban et al. reported different percentages for the main causes of MV, such as postoperative patients 10. Taken together, these disparities probably resulted in a less severely ill population with lower mortality rates 10,15.

The mortality of ARDS patients observed in our study was high. One reason for this high mortality rate in ARDS patients was the severity of illness, with a mean APACHE II score of 23.6. Furthermore, 42.5% of ARDS patients had failure of three or more organs. Additionally, in ARDS patients, the tidal volumes were higher than those suggested in the context of a lung-protective strategy, which reduces mortality in these patients 20. During the period of our study, a lung-protective strategy was not completely incorporated into our practice, which also could have affected the mortality rate. Another reason for discrepancy could be that in our study, we used a previously established criterion for the classification of ALI/ARDS 17, whereas mortality rates in other studies may have been influenced by the recent new Berlin definition of this syndrome 21. In Brazil in particular, using the Berlin definition, Azevedo et al. reported a hospital mortality rate of 52% 15. A systematic review found that ARDS mortality has remained static, at 44%, for observational studies since the 1994 consensus definition of this syndrome 22,23. For example, in the ALIEN study, the hospital mortality rate was 47.8% 24. In the setting of low-to-middle countries, best-practices strategies should be employed to reduce the mortality rates in ARDS patients.

Our finding that age was independently associated with mortality in MV patients has also been described in previous studies 8. In our study, COPD and pneumonia as causes of MV, in addition to VAP, were found to be negatively associated with mortality in these patients. COPD was also found to be protective by Esteban et al. 10. One possible explanation for the negative association of COPD and hospital mortality in the present study may be that such patients were less severely ill than those without COPD were; these patients presented fewer morbidities during the MV period (nonrespiratory system dysfunction). It should be noted that few patients had COPD exacerbation as a cause of MV and admission to the ICU in this study. Pneumonia as a cause of MV was also found to be negatively related to mortality, which may have been due to a lower percentage of failure of three or more organs in this group of patients. In two studies that examined risk factors for needing ventilatory support, pneumonia was not associated with mortality after the multivariate analysis 6,10. Studies of fatality rates comparing patients with and without VAP have generated contradictory results as to whether this condition increases mortality 10,. It should be noted that the present study was not designed to examine only VAP patients, but rather included a myriad of patients requiring MV. In addition, according to our analysis, VAP patients had a lower percentage of organ failure and of sepsis as a cause of MV compared with patients without VAP.

Our study showed a lower frequency of NIMV compared with IMV. Only 2.3% of patients remained on NIMV during the entire course of MV, which could be explained by our overwhelming patient demand for the ICU; in fact, many patients who needed NIMV remained in the emergency department or in the hospital ward. Our percentage of NIMV use was lower compared with percentages in other studies 15,19,28,29. For example, a recent Brazilian study had higher percentage of NIMV patients, which could have led to different outcomes 15. Additionally, the percentage of NIMV failure was higher in our study than in recent investigations 15,19,29, which may have been related to the difference in the severity of illness of the patients between the studies. More specifically, there was an important difference in the main reasons for the use of NIMV. Our study also found a higher percentage of patients with pneumonia, sepsis and ALI/ARDS and a small number of patients with a greater demonstrated benefit regarding COPD exacerbation and acute pulmonary edema 29,30,31.

The percentage of weaning failure in the present study was higher (29.4%) compared with percentages in the literature (5-20%) 15,32. Possible reasons may be related to (i) the fact that certain patients at high risk of weaning failure could not have used NIMV immediately after extubation and (ii) the lack of a weaning protocol. Although recognizing that a protocol would not necessarily be crucial, we think that it could have helped, considering that our ICU is a multidisciplinary unit with a heterogeneous patient population. Within the last few years, we have been using a weaning protocol including NIMV in high-risk patients, sharper surveillance regarding sedation, and physiotherapist participation in patients' weaning process. Indeed, recent data have shown that our percentage of weaning failure has dropped to 21% 32.

The limitations of our study include that (i) the data were obtained from one ICU, so the results may not be generalizable to other patients who require MV; (ii) the ICU was at a tertiary university hospital that admits patients with more severe illness/conditions, so the interpretation of the results may be different from that in other studies whose patients did not have similar disease severity; and (iii) comorbidities, such as neoplasia, AIDS, cirrhosis, and chronic renal failure, were not studied, so the influence of such conditions on the mortality rates of the studied population could not be estimated. Taking into account these limitations, to the best of our knowledge, this large study best describes the factors associated with mortality in ICU patients who required MV.

The hospital mortality rate of MV patients in our study was higher than rates reported in the literature, which may have been related to the severity of illness of the studied population. Furthermore, the factors associated with increased mortality included conditions present at the beginning of MV as well as conditions that occurred during mechanical support. The epidemiological aspects of MV patients explored in our investigation could enhance knowledge of such patients in South America. To the best of our knowledge, this large cohort study of MV patients on this continent may contribute to a more global understanding regarding the use of MV. In developing countries such as Brazil in particular, the limited availability of ICU beds results in the admission of patients with more severe illness, which may account for the higher mortality rates. These observations should be considered when comparing the mortality of such patients among countries under diverse socioeconomic conditions.

## AUTHOR CONTRIBUTIONS

Fialkow L designed the study, participated in the acquisition of the data, performed the statistical analyses of the data and wrote the manuscript. Farenzena M designed the study and participated in the acquisition of the data. Wawrzeniak IC conceived the study, analyzed and interpreted the data and wrote the manuscript. Brauner JS supervised the project, participated in the acquisition of the data, conceived the study, and analyzed and interpreted the data. Vieira SR supervised the project, conceived the study, participated in the acquisition of the data, and analyzed and interpreted the data. Vigo A performed the statistical analyses of the data. Bozzetti MC performed the statistical analyses of the data and wrote the manuscript. All authors contributed extensively to the work presented in this manuscript and took responsibility for reading and checking the text before submission.

## ACKNOWLEDGMENTS

We are thankful to Dr. Gregory P. Downey for his critical review of this manuscript.

Financial support was provided by FIPE-HCPA (Research and Events Support Fund at Hospital de Clínicas de Porto Alegre).

## Figures and Tables

**Table 1:** Characteristics of the Studied Patients on Admission to the Intensive Care Unit.

Characteristic	Number of Patients Mechanically Ventilated
Age, median±SD	57.2±18
Gender, males	582 (52%)
APACHE II score, mean±SD	22.6±8.3
Medical/Surgical	771 (69%)/344 (31%)
Source of admission	
Hospital ward	625 (56%)
Emergency department	278 (25%)
Other hospitals	212 (19%)
Reason for the initiation of mechanical ventilation^1^	
Sepsis	466 (41.8%)
Shock	421 (37.8%)
Pneumonia	415 (37%)
ALI	167 (15%)
ALI without ARDS	40 (3.6%)
ARDS	127 (11.4%)
Neurological condition	
Stroke	79 (7.1%)
Neuromuscular disease	15 (1.3%)
Other neurological condition	32 (3%)
COPD	60 (5.4%)
Cardiac arrest	67 (6.0%)
Asthma	16 (1.6%)
Other	14 (1.2%)

APACHE II = Acute Physiology and Chronic Health Evaluation II; ALI = acute lung injury; ARDS = acute respiratory distress syndrome; COPD = chronic obstructive pulmonary disease; SD = standard deviation; ^1^ more than one reason for initiation of mechanical ventilation was allowed.

**Table 2:** Ventilator Modes and Monitored Variables on Days 1, 2 and 3 (Mean Values) of Invasive Mechanical Ventilation.

	Overall (n=976)^1^	COPD (n=56)^a^	ARDS (n=263)^a^
Ventilator mode, no. (%)			
ACP	715 (73)	37 (66)	200 (76)
PSV	195 (20)	14 (25)	46 (17.5)
CPAP	29 (3)	3 (5)	9 (3.5)
ACV	22 (2.5)	2 (4)	7 (2.5)
SIMV	15 (1.5)	0 (0)	7 (2.5)
Monitored variable, mean±SD			
Peak pressure, cm H_2_O	25.5±8.1	24±4.8	28.5±8.2
Plateau pressure, cm H_2_O	21.5±5.8	20.2±4.6	23.6±6.4
Tidal volume, ml/kg (PBW)	10.5±3.3	10±3	9±3.3
Respiratory rate, breaths/min	20.1±4.1	19.3±3.8	21.2±4.4
PaO_2_/FiO_2_	298.8±155	292.4±118	169±111.3
PEEP, cm H_2_O	6.6±2.5	5.8±1.5	9±4

COPD = chronic obstructive pulmonary disease; ARDS = acute respiratory distress syndrome; ACP = assisted/controlled pressure ventilation; ACV = assisted/controlled volume ventilation; PSV = pressure support ventilation; CPAP = continuous positive airway pressure; SIMV = synchronized intermittent mandatory ventilation; PBW = predicted body weight; PEEP = positive end-expiratory pressure; SD = standard deviation; ^1^ the percentages of missing data for all patients and for the COPD and ARDS groups in particular were 12%, 11%, and 14%, respectively; ^2^ refers to non-COPD and non-ARDS patients.

**Table 3:** Duration of Mechanical Ventilation and Length of Stay in the Intensive Care Unit and in the Hospital in the Studied Patients.

	Other Patients^2^ (n=748)	COPD (n=60)	ARDS (n=307)	*p*-value*
Duration of MV^1^	9.3±7.3	8.9±5.5	12.1±8.7	<0.01**
LOS in ICU^1^	14.6±11.5	14.3±10	15.6±11.6	0.19
LOS in hospital^1^	24.7±22.9	24.3±18.1	22.3±21	0.12

MV = mechanical ventilation; LOS = length of stay; COPD = chronic obstructive pulmonary disease; ARDS = acute respiratory distress syndrome;

1 days;

2 refers to non-COPD and non-ARDS patients;

**<?ENTCHAR ast?>:** *p*-value for ANOVA;

**<?ENTCHAR ast?><?ENTCHAR ast?>:** *p*<0.001 for ANOVA (and *p*<0.05 for the Tukey test for the following comparisons: COPD *vs*. ARDS patients and other patients *vs*. ARDS patients).

**Table 4:** Severity of Illness and Hospital Mortality According to the Number of Organs with Failure.

Group	n	Age*	APACHE II Score*	Hospital Mortality (%)**	HR (95% CI)**
ARF+0	138	57.5±17.8	19.0±7.2	21.0%	1.0
ARF+1	248	57.1±19.2	19.9±7.4	37.1%	1.91 (1.26-2.89)
ARF+2	285	58.7±17.3	22.7±7.6	54.4%	3.35 (2.26-4.99)
ARF+3	249	58.5±17.3	24.9±8.4	64.7%	4.19 (2.82-6.23)
ARF+4	139	54.0±17.5	26.6±9.2	69.8%	4.62 (3.05-6.99)
ARF+5	56	52.4±19.9	25.3±7.3	73.2%	5.24 (3.25-8.43)

APACHE II = Acute Physiology and Chronic Health Evaluation II; ARF = acute respiratory failure; HR = hazard ratio; CI = confidence interval;

**<?ENTCHAR ast?>:** mean±standard deviation;

**<?ENTCHAR ast?><?ENTCHAR ast?>:** *p*-value for linear trend: hospital mortality (*p*<0.001) and HR (*p*<0.001).

**Table 5:** Statistical Analysis of the Factors Associated with Mortality in Mechanical Ventilation Patients.

Variable	Univariable		Multivariable	
	HR (95% CI)*	*p*-value**	HR (95% CI)*	*p*-value**
Age (years)	1.07 (1.002-1.012)	0.004	1.01 (1.004-1.014)	<0.001
APACHE II score	1.01 (1.007-1.02)	<0.0001	1.01 (1.002-1.014)	<0.005
Chronic use of corticosteroids	0.72 (0.53-0.97)	0.03		
Failure previous to MV				
Cardiovascular	1.42 (1.20-1.67)	<0.0001		
Coagulopathic	1.46 (1.21-1.77)	<0.0001		
Hepatic	1.26 (1.02-1.57)	0.03		
Renal	1.34 (1.14-1.59)	0.0005	1.29 (1.06-1.56)	0.011
Neurological	1.13 (0.94-1.35)	0.17	1.25 (1.031.52)	0.024
Cause of MV initiation				
COPD	0.42 (0.25-0.69)	0.0006	0.58 (0.35-0.98)	0.042
ALI/ARDS	1.33 (1.08-1.65)	0.008	1.38 (1.08-1.77)	0.009
Sepsis	1.47 (1.25-1.73)	<0.0001	1.33 (1.10-1.61)	0.003
Pneumonia	0.88 (0.74-1.05)	0.15	0.78 (0.65-0.95)	0.013
Asthma	0.43 (0.16-1.15)	0.09		
Condition occurring during MV				
Sepsis	1.48 (1.22-1.79)	<0.0001	1.53 (1.23-1.90)	<0.001
VAP	0.70 (0.53-0.91)	0.009	0.62 (0.46-0.83)	0.001
ALI/ARDS	1.42 (1.19-1.71)	0.0001	1.31 (1.07-1.61)	<0.010
Failure				
Cardiovascular	1.54 (1.28-1.85)	<0.0001	1.32 (1.07-1.62)	≤0.009
Coagulopathic	1.41 (1.16-1.72)	0.0006		
Hepatic	1.91 (1.48-2.48)	<0.0001	1.67 (1.26-2.21)	≤0.001
Renal	1.86 (1.57-2.22)	<0.0001	1.75 (1.42-2.15)	<0.001

ALI/ARDS = acute lung injury/acute respiratory distress syndrome; APACHE II = Acute Physiology and Chronic Health Evaluation II; COPD = chronic obstructive pulmonary disease; MV = mechanical ventilation; VAP= ventilator-associated pneumonia;

**<?ENTCHAR ast?>:** hazard ratio (HR) and 95% confidence interval (CI) for the HR;

**<?ENTCHAR ast?><?ENTCHAR ast?>:** *p*-value for the analysis. In the final multivariate model, variables with *p*>0.05 were not included.

## References

[1] Slutsky AS (2015). History of Mechanical Ventilation. From Vesalius to Ventilator-induced Lung Injury. Am J Respir Crit Care Med.

[2] Rittayamai N, Katsios CM, Beloncle F, Friedrich JO, Mancebo J, Brochard L (2015). Pressure-Controlled vs Volume-Controlled Ventilation in Acute Respiratory Failure: A Physiology-Based Narrative and Systematic Review. Chest.

[3] Vincent JL, Akca S, De Mendonca A, Haji-Michael P, Sprung C, Moreno R (2002). The epidemiology of acute respiratory failure in critically ill patients(*). Chest.

[4] Vasilyev S, Schaap RN, Mortensen JD (1995). Hospital survival rates of patients with acute respiratory failure in modern respiratory intensive care units. An international, multicenter, prospective survey. Chest.

[5] Luhr OR, Antonsen K, Karlsson M, Aardal S, Thorsteinsson A, Frostell CG (1999). Incidence and mortality after acute respiratory failure and acute respiratory distress syndrome in Sweden, Denmark, and Iceland. The ARF Study Group. Am J Respir Crit Care Med.

[6] Behrendt CE (2000). Acute respiratory failure in the United States: incidence and 31-day survival. Chest.

[7] Wunsch H, Linde-Zwirble WT, Angus DC, Hartman ME, Milbrandt EB, Kahn JM (2010). The epidemiology of mechanical ventilation use in the United States. Crit Care Med.

[8] Carson SS, Cox CE, Holmes GM, Howard A, Carey TS (2006). The changing epidemiology of mechanical ventilation: a population-based study. J Intensive Care Med.

[9] Needham DM, Bronskill SE, Sibbald WJ, Pronovost PJ, Laupacis A (2004). Mechanical ventilation in Ontario, 1992-2000: incidence, survival, and hospital bed utilization of noncardiac surgery adult patients. Crit Care Med.

[10] Esteban A, Anzueto A, Frutos F, Alia I, Brochard L, Stewart TE (2002). Characteristics and outcomes in adult patients receiving mechanical ventilation: a 28-day international study. JAMA.

[11] Linko R, Okkonen M, Pettila V, Perttila J, Parviainen I, Ruokonen E (2009). Acute respiratory failure in intensive care units. FINNALI: a prospective cohort study. Intensive Care Med.

[12] Lewandowski K, Metz J, Deutschmann C, Preiss H, Kuhlen R, Artigas A (1995). Incidence, severity, and mortality of acute respiratory failure in Berlin, Germany. Am J Respir Crit Care Med.

[13] Flaatten H, Gjerde S, Guttormsen AB, Haugen O, Hoivik T, Onarheim H (2003). Outcome after acute respiratory failure is more dependent on dysfunction in other vital organs than on the severity of the respiratory failure. Crit Care.

[14] Tomicic V, Espinoza M, Andresen M, Molina J, Calvo M, Ugarte H (2008). [Characteristics and factors associated with mortality in patients receiving mechanical ventilation: first Chilean multicenter study]. Rev Med Chil.

[15] Azevedo LC, Park M, Salluh JI, Rea-Neto A, Souza-Dantas VC, Varaschin P (2013). Clinical outcomes of patients requiring ventilatory support in Brazilian intensive care units: a multicenter, prospective, cohort study. Crit Care.

[16] Vincent JL, de Mendonca A, Cantraine F, Moreno R, Takala J, Suter PM (1998). Use of the SOFA score to assess the incidence of organ dysfunction/failure in intensive care units: results of a multicenter, prospective study. Working group on &quot;sepsis-related problems&quot; of the European Society of Intensive Care Medicine. Crit Care Med.

[17] Bernard GR, Artigas A, Brigham KL, Carlet J, Falke K, Hudson L (1994). The American-European Consensus Conference on ARDS. Definitions, mechanisms, relevant outcomes, and clinical trial coordination. Am J Respir Crit Care Med.

[18] American Thoracic S, Infectious Diseases Society of A (2005). Guidelines for the management of adults with hospital-acquired, ventilator-associated, and healthcare-associated pneumonia. Am J Respir Crit Care Med.

[19] Esteban A, Ferguson ND, Meade MO, Frutos-Vivar F, Apezteguia C, Brochard L (2008). Evolution of mechanical ventilation in response to clinical research. Am J Respir Crit Care Med.

[20] Ventilation with lower tidal volumes as compared with traditional tidal volumes for acute lung injury and the acute respiratory distress syndrome (2000). The Acute Respiratory Distress Syndrome Network. N Engl J Med.

[21] Force ADT, Ranieri VM, Rubenfeld GD, Thompson BT, Ferguson ND, Caldwell E (2012). Acute respiratory distress syndrome: the Berlin Definition. JAMA.

[22] Phua J, Badia JR, Adhikari NK, Friedrich JO, Fowler RA, Singh JM (2009). Has mortality from acute respiratory distress syndrome decreased over time?: A systematic review. Am J Respir Crit Care Med.

[23] Rubenfeld GD, Caldwell E, Peabody E, Weaver J, Martin DP, Neff M (2005). Incidence and outcomes of acute lung injury. N Engl J Med.

[24] Villar J, Blanco J, Anon JM, Santos-Bouza A, Blanch L, Ambros A (2011). The ALIEN study: incidence and outcome of acute respiratory distress syndrome in the era of lung protective ventilation. Intensive Care Med.

[25] Ranes JL, Gordon SM, Chen P, Fatica C, Hammel J, Gonzales JP (2006). Predictors of long-term mortality in patients with ventilator-associated pneumonia. Am J Med.

[26] Melsen WG, Rovers MM, Bonten MJ (2009). Ventilator-associated pneumonia and mortality: a systematic review of observational studies. Crit Care Med.

[27] Myny D, Depuydt P, Colardyn F, Blot S (2005). Ventilator-associated pneumonia in a tertiary care ICU: analysis of risk factors for acquisition and mortality. Acta Clin Belg.

[28] Girault C, Briel A, Hellot MF, Tamion F, Woinet D, Leroy J (2003). Noninvasive mechanical ventilation in clinical practice: a 2-year experience in a medical intensive care unit. Crit Care Med.

[29] Schettino G, Altobelli N, Kacmarek RM (2008). Noninvasive positive-pressure ventilation in acute respiratory failure outside clinical trials: experience at the Massachusetts General Hospital. Crit Care Med.

[30] Brochard L, Mancebo J, Wysocki M, Lofaso F, Conti G, Rauss A (1995). Noninvasive ventilation for acute exacerbations of chronic obstructive pulmonary disease. The New England Journal of Medicine.

[31] Masip J, Betbese AJ, Paez J, Vecilla F, Canizares R, Padro J (2000). Non-invasive pressure support ventilation versus conventional oxygen therapy in acute cardiogenic pulmonary oedema: a randomised trial. Lancet.

[32] Epstein SK (2009). Weaning from ventilatory support. Curr Opin Crit Care.

[33] Teixeira C, Maccari JG, Vieira SR, Oliveira RP, Savi A, Machado AS (2012). Impact of a mechanical ventilation weaning protocol on the extubation failure rate in difficult-to-wean patients. Jornal brasileiro de pneumologia : publicacao oficial da Sociedade Brasileira de Pneumologia e Tisilogia.

